# The impact of age-related cataracts on colour perception, postoperative recovery and related spectra derived from test of hue perception

**DOI:** 10.1186/s12886-019-1057-6

**Published:** 2019-02-20

**Authors:** Mingxin Ao, Xuemin Li, Weiqiang Qiu, Zhiqiang Hou, Jie Su, Wei Wang

**Affiliations:** 1 Department of Ophthalmology, Peking University Third Hospital, Beijing Key Laboratory of Restoration of Damaged Ocular Nerve, Number Forty-Nine North Garden Road Haidian District, Beijing, 86100191 China; 2 Beijing Key Laboratory of Restoration of Damaged Ocular Nerve, Number Forty-Nine North Garden Road Haidian District, Beijing, 86100191 China

**Keywords:** Colour vision, Age-related cataract, Intraocular lens, Clinical outcome, Farnsworth-Munsell 100-hue test

## Abstract

**Background:**

Cataract patients were always excluded from studies on ageing of colour vision; thus, effect of age-related cataracts on deterioration of colour perception has not been analysed. In present study, impacts of age-related cataracts on colour discrimination, postoperative recovery and related spectra were investigated.

**Methods:**

In this cohort study, thirty age-related cataract patients scheduled for binocular surgery and 30 elderly volunteers were enrolled. Colour discrimination under photopic (1000 lx) and mesopic (40 lx) conditions was evaluated with Farnsworth-Munsell 100-hue test. The total error score (TES) and partial error score (PES) were calculated.

**Results:**

Preoperatively, the TES in the patient group was 129.7 ± 59.5 at 1000 lx and 194.6 ± 74.5 at 40 lx, exhibiting worse discrimination than the volunteer group (TES_1000lux_ = 71.5 ± 37.5 and TES_40lux_ = 113.1 ± 38.8, *p* ≤ 0.001). Inferior perception were detected in the yellow to green-yellow (Y-GY), green-yellow to green (GY-G), green to blue-green (G-BG) and blue-green to blue (BG-B) colour bands (*p* ≤ 0.003), corresponding to the 470 nm–580 nm range of the visible light spectrum. Under mesopic conditions, the impact expanded to all colour bands except for yellow-red to yellow (YR-Y). Postoperatively, the TES in the patient group were 80.4 ± 62.4 at 1000 lx and 112.0 ± 85.2 at 40 lx, which were lower than those of the preoperative phase (*p* ≤ 0.001) but similar to those of the volunteer group (*p ≥* 0.505). Postoperative improvement occurred in the Y-GY, GY-G and G-BG colour bands (490 nm to 580 nm) at 1000 lx (*p* ≤ 0.001) and shifted to the Y-GY, GY-G, G-BG and BG-B colour bands (470 nm to 580 nm) at 40 lx (*p* ≤ 0.001). Deterioration of hue perception for decrement of illumination was detected in the red to yellow-red (R-YR), Y-GY, G-BG, BG-B, blue to purple-blue (B-PB) and red-purple to red (RP-R) colour bands (450 nm to 500 nm) in the volunteer group (*p* ≤ 0.002) and the R-YR, G-BG, BG-B, B-PB, PB-P and red-purple to red (RP-R) colour bands (from the short-wavelength end to 500 nm) in the patient group preoperatively (*p* ≤ 0.001).

**Conclusions:**

Phacoemulsification could effectively rebuild colour perception in patients with age-related cataract. The postoperative benefits were most significant in colour bands corresponding with spectrum from 470 nm to 580 nm.

**Electronic supplementary material:**

The online version of this article (10.1186/s12886-019-1057-6) contains supplementary material, which is available to authorized users.

## Background

Colour vision is an essential part of visual function. The perception of colour encompasses light transmittance via the optical system (i.e., cornea, pupil and crystalline lens), signal initiation in photoreceptors, transduction of post-receptor channels and decoding in the visual cortex [1–4]. All elements of human colour vision are significantly affected by ageing [5–7]. Based on electrophysiological measurements [8, 9] and psychophysical tasks [6, 10–15], investigators have confirmed that due to ageing, colour discrimination primarily declined along the yellow-blue axis. In these studies, elderly individuals with cataracts were excluded because cataracts severely reduce contrast in retinal images, thereby affecting colour constancy prerequisites [16–18]. Deteriorations in colour perception resulting from age-related cataracts have yet to be analysed. After cataract surgery, the pseudophakic eye is a model free from the effects of lenticular senescence. Intensive research on colour discrimination before and after cataract surgery is essential to current knowledge about colour perception and will provide valuable insight on the effects of ageing on colour vision.

By testing for achromatic settings [19, 20] and minimum motion task [21], studies have confirmed differences in perceiving short-wavelength light between patients with senile cataracts and patients undergoing cataract surgery. However, due to the lack of comparisons between the lens of middle-aged volunteers and mildly aged lens, the status of colour vision after cataract surgery has not been discussed in the context of postoperative benefits; instead, researchers have simply named this condition as cyanopsia [22]. Past studies have only measured sensitivity to lights within specific spectral ranges. Changes in colour discrimination over the entire visible spectrum have not been comprehensively assessed; as such, data on postoperative benefits in colour vision are lacking. The range of spectra corresponding to benefits in colour perception may be an important factor affecting the optimal filtering property of intraocular lens in balancing macular protection and visual quality. Clinical studies should be designed to determine enhanced colour perception after cataract surgery and to identify related light spectra.

The purpose of our study is to assess colour discrimination due to age-related cataracts and phacoemulsification surgery. By using the Farnsworth-Munsell (FM) 100-hue test, changes in colour discrimination and corresponding spectra were examined. Our findings suggested that lens opacity and postoperative benefits on colour vision were most significant in colour bands corresponding to light wavelengths from 470 nm to 580 nm.

## Methods

### Patients and study population

A cohort study was conducted. In the patient group, participants were recruited from patients who were referred for cataract surgery in the Department of Ophthalmology of Peking University Third Hospital from January 2014 through April 2015. The inclusion criteria included diagnosis of binocular age-related cataract, eligibility for bilateral surgery, a d corrected distant visual acuity (CDVA) difference between right eye and left eye of less than 2 lines, compatibility of lens opacity classification between right eye and left eye, between 60 and 80 years of age, in good general health with no ocular pathologic features other than age-related cataract and no history of colour-vision deficiency. Exclusion criteria included previous ocular surgery or laser treatment, a postoperative CDVA worse than 20/25, previous or current use of medications known to cause colour-vision deficiency, failure to pass a colour-screening test (Ishihara plate test), intraoperative or postoperative complications and detection of macular disease in fundus examination at preoperative phase or postoperative phase. To exclude known impacts on colour vision [23], subjects with diabetes or subjects with impaired fasting glucose were excluded from the study. A control group was formed using elderly volunteers, who came to our hospital for presbyopic prescriptions. Other than a crystalline lens without cortical or posterior subcapsular opacity in the pupil area and graded less than NO-3 or NC-3 (no more than NO-2.9 or NC-2.9) [24, 25] by the LOCS-III criteria [26] under slit-lamp examination, the inclusion and exclusion criteria for the control group were the same as those for the patient group. For the critical criteria of lens opacity, the age range of control group was broadened to 50 to 80 years. This research was approved by the Peking University Third Hospital Medicine Ethics Committee (IRB00006761–2012046) and was conducted in accordance with the Declaration of Helsinki. Informed written consent was obtained from each participant.

The sample size was calculated by PASS software, version 2011 (NCSS, LLC, Kaysville, UT, USA). The TES data of the first 15 cases in the patient group at the very beginning (details in Additional files 1, 2, 5 and 6) were used in the calculation for sample size. A paired design was employed (with significance level alpha of 0.05 and power level of 90%), which suggested a minimum sample size of 30 cases under photopic condition and 22 cases under mesopic condition for the detection of changes in overall colour perception.

### Surgical procedures and ophthalmological examinations

Intervention was standard minimal incision phacoemulsification with topical anaesthesia. An aspherical, monofocal, non-yellow-tinted IOL (Tecnis Fordable Acrylic Intraocular Lenses, ZA9003, Abbott Medical Optics, Inc.) was inserted through a private injector and cartridge into the capsular bag. After surgery, the patients were treated with a combination of levofloxacin (Gravit) eye drops and prednisolone acetate (Pred Forte) ophthalmic suspension 4 times per day for 1 week, the frequency of which was then gradually reduced. All cataract surgeries were performed by one experienced surgeon (W.W.).

Refraction, Snellen CDVA under standard condition with logarithm of the minimum angle of resolution (logMAR) conversion, slit-lamp biomicroscopy, fundoscopy, and non-contacted tonometry were performed at all visits (i.e., baseline and 1-day, 1-week, 1-month and 3-month, postoperatively). Cataracts were classified and graded according to LOCS-III criteria under slit-lamp examination at baseline under mydriasis by 0.5% tropicamide. All subjects were examined by two experienced physicians (ZQ.H. and WQ.Q.).

### Colour perception test

The FM 100-hue test has a standard eighty-five stimuli of different hues, i.e., the same saturation and coverage as the whole visible spectrum [27]. This test can be used to assess colour vision based on a total error score or the deterioration of colour perception to special hues or along single colour axes based on partial error score. The FM 100-hue test is a simple and relatively sensitive test and has been widely used in examining the effects of achromatopsia [28], optic neuropathy [29, 30], diabetic retinopathy [23, 31] and IOL on colour perception [32]. In the present study, colour discrimination was measured using the FM 100-hue test. Colour vision and aptitude abnormalities were detected by the subject’s ability to place removable colour reference caps in order of hue. The colour discrimination test was carried out at baseline and 1-month, postoperatively. Patients rested for 15 min before taking the FM 100-hue test. The test was performed binocularly with spectacle correction, if necessary. The test procedure was implemented according to manufacturer instructions. Patients took the entire test under both mesopic and photopic conditions with an interval of 10 min between conditions. The photopic condition was set up with an illumination of approximately 1000 lx and the mesopic condition with an illumination of approximately 40 lx.

The error scores were calculated by FM 100-hue test scoring software (version 3.0). A total error score (TES) and the score of every colour cap test were recorded under each condition. The TES represents a general colour perception capability. Partial error scores (PES) for 10 colour bands were calculated to assess hue discrimination. The colour bands were: red to yellow-red (R-YR), yellow-red to yellow (YR-Y), yellow to green-yellow (Y-GY), green-yellow to green (GY-G), green to blue-green (G-BG), blue-green to blue (BG-B), blue to purple-blue (B-PB), purple-blue to purple (PB-P), purple to red-purple (P-RP) and red-purple to red (RP-R). To explore the relationship between colour perception and sensitivity to visible light, colour bands in the Munsell system were correlated with the Commission Internationale de L’Eclairage (CIE) chromaticity diagram, and wavelengths related to surface colour were identified. According to the CIE diagram and past studies that approximated light wavelength ranges with Munsell Farnsworth’s diagram [27], the R-YR, YR-Y, Y-GY, GY-G, G-BG, BG-B, B-PB and PB-P colour bands corresponded with wavelengths of end of long wavelength to 590 nm, 590 nm to 580 nm, 580 nm to 560 nm, 560 nm to 500 nm, 500 nm to 490 nm, 490 nm to 470 nm, 470 nm to 450 nm and 450 nm to the end of short wavelength, respectively. As the complementary colours of green to yellowish green, the P-RP and RP-R colour bands correspond to 560 nm to 500 nm.

### Statistical analysis

Statistical analysis was performed using SPSS software, version 16.0 (SPSS, Inc., Chicago, IL, USA). Data distribution normality was checked by the Kolmogorov-Smirnoff-Lillefors test. Descriptive statistics for continuous variables normally distributed were reported as the means ± standard deviations (*SD*). Variables not normally distributed were described as median and range. Frequency distribution and percentages were used for nominal variables. Continuous variables were compared using a two-tailed 2-sample *t*-test or two-tailed paired *t-*test when data displayed normal distributions; the Wilcoxon rank-sum test was used for non-normal distributions. Pearson’s chi-square test was adopted to analyse the proportions of categorical data. Pearson correlation analysis was employed to detect potential correlations between lens opacity and colour perception. The level of statistical significance (the alpha level) was *p* < 0.05. For PES data, this value was 0.0045 for Bonferroni’s correction (0.05 of 11) [33, 34].

## Results

### Demographic characteristics of participants

A total of 30 patients (18 females and 12 males with the age of 69.6 ± 7.1) were recruited from 426 candidates invited to a screening for the study (7%). Preoperative CDVA was 0.39 ± 0.17 for the right eye and 0.42 ± 0.20 for the left eye, which was comparable between the two eyes (Wilcoxon signed ranks test, *p* = 0.261).

Two physicians (ZQ.H. and WQ.Q.) classified the lens opacity, and a mean of the two graders’ classification was used. The mean grading score of lens opacity by the LOCS-III criteria for the right eye was 3.50 ± 0.70 for nuclear colour (NC), 3.30 ± 0.60 for nuclear opalescence (NO), 3.49 ± 0.86 for cortical opalescence (C) and 0.84 ± 0.81 for posterior subcapsular opalescence (P). For the left eye, the score was 3.51 ± 0.67 for NC, 3.40 ± 0.72 for NO, 3.28 ± 0.92 for C and 0.70 ± 0.69 for P. The results between the two observers were consistent for right eye NC (paired *t*-test, *p* = 0.313), NO (paired *t*-test, *p* = 0.740), C (paired *t*-test, *p* = 0.508), P (paired *t*-test, *p* = 0.879) and left eye NC (paired *t*-test, *p* = 0.100), NO (paired *t*-test, *p* = 0.910), C (paired *t*-test, *p* = 0.117), and P (paired *t*-test, *p* = 0.823). These results suggested that cortical and nuclear cataracts were the main cataract types in our study. Scores of LOCS-III were compatible between the two eyes in terms of NC (paired *t*-test, *p* = 0.753), NO (paired *t*-test, *p* = 0.233), C (paired *t*-test, *p* = 0.222) and P (paired *t*-test, *p* = 0.284).

Thirty volunteers (22 females and 8 males) with an average age of 61.1 ± 7.5 years were enrolled in the volunteer group. No differences were found between the two groups in the distribution of sex (Pearson’s chi-square test, *p* = 0.273). For the inclusion criteria of crystalline lens, graded less than NO-3 or NC-3 (no more than NO-2.9 or NC-2.9), the subjects in the volunteer group were significantly younger than those in the patient group (2-sample *t* test, *p* ≤ 0.001).

### Effects of age-related cataracts on colour perception

Age-related cataracts severely interfered with colour perception in elderly people. Under photopic condition, colour perception was significantly worse in the patient group (TES of 129.7 ± 59.5) than in the volunteer group (TES of 71.5 ± 37.5, 2-sample *t* test, *p* ≤ 0.001). Hue perception investigations indicated a significantly higher PES in the R-YR, Y-GY, GY-G, G-BG, BG-B and P-RP colour bands in the patient group (2-sample *t* test, *p* ≤ 0.003). The PES values under photopic condition are presented in Table 1. Under mesopic condition, TES in the patient group was 194.6 ± 74.5, which was significantly higher than that in the volunteer group (113.1 ± 38.8, 2-sample *t* test, *p* ≤ 0.001). Hue discrimination inferiority expanded to most colour bands around the FM circuit except for the YR-Y band (2-sample *t* test, *p* = 0.079; Table 2). These results suggested that the impact of age-related cataracts on hue discrimination was significant in surface colours corresponding to light wavelengths from 470 to 580 nm. This impact shifts to colour bands associated with light wavelengths of 580 nm to the short-wavelength end under mesopic condition. The error scores of each patient at preoperative phase are listed in Additional files 1 and 2. The error scores of each volunteer in the volunteer group are listed in Additional files 3 and 4.

**Table 1:** Error scores in colour bands of FM 100-hue test under photopic condition

Colour band	Partial error score Mean ± *SD*			*p* value		
	Control	Patient -pre	Patient -post	Control vs. patient-pre	Control vs. patient-post	Patient -pre vs. -post
R-YR	9.3 ± 6.7	16.7 ± 10.7	9.1 ± 8.4	0.002^*^	0.946	0.001^*^
YR-Y	2.7 ± 3.3	5.3 ± 5.3	5.6 ± 5.4	0.026	0.016	0.766
Y-GY	8.9 ± 5.8	15.7 ± 7.9	8.2 ± 7.5	0.000^*^	0.674	0.000^*^
GY-G	13.8 ± 8.1	23.5 ± 9.9	14.7 ± 12.0	0.000^*^	0.716	0.000^*^
G-BG	14.7 ± 9.4	28.0 ± 15.0	15.2 ± 14.6	0.000^*^	0.875	0.000^*^
BG-B	9.4 ± 8.0	17.5 ± 10.7	11.4 ± 11.4	0.002^*^	0.428	0.013
B-PB	5.1 ± 3.9	8.2 ± 5.8	6.9 ± 6.6	0.019	0.195	0.322
PB-P	1.7 ± 3.2	4.1 ± 4.1	4.2 ± 5.2	0.018	0.031	0.901
P-RP	3.4 ± 3.5	8.1 ± 7.3	4.7 ± 5.5	0.003^*^	0.267	0.021
RP-R	10.5 ± 8.1	18.9 ± 14.3	9.6 ± 9.1	0.008	0.688	0.001^*^

**Table 2:** Error scores in colour bands of FM 100-hue test under mesopic condition

Colour band	Partial error score Mean ± *SD*			*p* value		
	Control	Patient -pre	Patient -post	Control vs. patient-pre	Control vs. patient-post	Patient -pre vs. -post
R-YR	16.0 ± 7.5	26.1 ± 13.7	15.3 ± 12.1	0.001^*^	0.808	0.000^*^
YR-Y	5.6 ± 5.3	9.0 ± 8.4	6.6 ± 5.5	0.067	0.477	0.112
Y-GY	12.5 ± 5.8	19.3 ± 10.5	10.0 ± 8.6	0.003^*^	0.206	0.000^*^
GY-G	18.4 ± 8.4	28.4 ± 12.4	15.9 ± 12.3	0.001^*^	0.375	0.000^*^
G-BG	23.0 ± 11.2	40.8 ± 17.1	24.4 ± 22.5	0.000^*^	0.756	0.000^*^
BG-B	18.7 ± 9.6	32.7 ± 16.4	17.8 ± 17.0	0.000^*^	0.809	0.000^*^
B-PB	8.1 ± 4.1	14.5 ± 7.9	9.6 ± 9.0	0.000^*^	0.410	0.005
PB-P	3.3 ± 3.3	9.0 ± 7.0	5.5 ± 6.8	0.000^*^	0.112	0.057
P-RP	4.3 ± 3.5	10.9 ± 7.9	6.1 ± 6.6	0.000^*^	0.204	0.002^*^
RP-R	17.5 ± 9.8	28.7 ± 15.4	14.7 ± 14.3	0.001^*^	0.386	0.000^*^

Under photopic condition (Table 3), NC classification was correlated with the BG-B error score (*r* = 0.443, *p* = 0.014) and NO classification was correlated with the BG-B (*r* = 0.451, *p* = 0.012) and B-PB (*r* = 0.384, *p* = 0.036) error scores. Under mesopic condition (Table 4), NC classification was correlated with the G-BG (*r* = 0.463, *p* = 0.010), BG-B (*r* = 0.466, *p* = 0.009) and B-PB (*r* = 0.489, *p* = 0.006) error scores and NO classification was correlated with TES (*r* = 0.416, *p* = 0.022) and the G-BG (*r* = 0.499, *p* = 0.005), BG-B (*r* = 0.416, *p* = 0.022), P-RP (*r* = 0.475, *p* = 0.008) and RP-R (*r* = 0.485, *p* = 0.007) error scores.

**Table 3:** Correlation coefficient in Pearson correlation analysis between error score in FM 100-Hue and lens opacity classification by LOCS III under photopic condition

Error score	Lens opacity classification by LOCS III			
	NC	NO	C	PS
TES	0.307	0.305	0.293	−0.175
R-YR	0.149	0.012	0.302	−0.041
YR-Y	0.177	0.091	−0.056	−0.087
Y-GY	0.242	0.289	0.119	−0.081
GY-G	0.293	0.278	0.081	−0.132
G-BG	0.306	0.280	0.259	− 0.180
BG-B	0.443^*^	0.451^*^	0.228	−0.307
B-PB	0.288	0.384^*^	0.309	−0.175
PB-P	0.012	0.051	0.332	0.073
P-RP	0.025	0.218	0.253	−0.154
RP-R	0.113	0.133	0.275	−0.100

**Table 4:** Correlation coefficient in Pearson correlation analysis between error score in FM 100-Hue and lens opacity classification by LOCS III under mesopic condition

Error score	Lens opacity classification by LOCS III			
	NC	NO	C	PS
TES	0.336	0.416^*^	0.028	−0.116
R-YR	0.197	0.338	0.148	−0.012
YR-Y	0.119	0.093	−0.081	0.158
Y-GY	−0.112	−0.014	− 0.124	−0.119
GY-G	0.078	0.189	−0.175	−0.113
G-BG	0.463^*^	0.499^*^	−0.040	−0.117
BG-B	0.466^*^	0.416^*^	0.051	−0.083
B-PB	0.489^*^	0.315	0.002	−0.173
PB-P	−0.214	−0.140	0.117	−0.151
P-RP	0.222	0.475^*^	0.143	−0.084
RP-R	0.348	0.485^*^	0.174	−0.083

Values are presented as the means ± standard deviation. For comparisons between the volunteer group and the patient group, a 2-sample t-test was employed for continuous variables; for intra-individual comparisons between the preoperative phase and the postoperative phase, a paired t-test was used for continuous variable. ^*^ indicates significance after Bonferroni’s correction.

Values are presented as the means ± standard deviation. For comparisons between the volunteer group and the patient group, a 2-sample t-test was employed for continuous variables; for intra-individual comparisons between the preoperative phase and the postoperative phase, a paired t-test was used for continuous variable. ^*^ indicates significance after Bonferroni’s correction.

Results in the table are correlation coefficients, ^*^ indicates significance (*p* < 0.05) in Pearson correlation analyses.

Results in the table are correlation coefficients, ^*^ indicates significance (*p* < 0.05) in Pearson correlation analyses.

### Postoperative recovery of colour perception

After cataract surgery, general colour perception (TES_photopic_ = 80.4 ± 62.4 and TES_mesopic_ = 112.0 ± 85.2) significantly improved compared with that at preoperative phase (paired *t*-test, *p* ≤ 0.001) and recovered to a level comparable with that of the volunteer group (2-sample *t* test, *p*_photopic_ = 0.505 and *p*_mesopic_ = 0.951). There were no significant differences in hue discrimination between the patient group at postoperative phase and the volunteer group according to the PES of the 10 colour bands under photopic or mesopic conditions (2-sample *t* test, *p* ≥ 0.021). The results demonstrated that phacoemulsification could effectively restore colour discrimination in patients with age-related cataracts.

In the patient group, further analyses were conducted to locate colour bands and the associated spectra associated with postoperative changes. Postoperative photopic hue perception changes were detected in the R-YR, Y-GY, GY-G, G-BG and RP-R colour bands (paired *t*-test, *p* ≤ 0.001). Under mesopic condition, postoperative hue perception changes expanded to the R-YR, Y-GY, GY-G, G-BG, BG-B, P-RP and RP-R colour bands (paired *t*-test, *p* ≤ 0.002). This analysis revealed that a postoperative hue perception improvement occurred in colour bands correlated with light wavelengths from 490 nm to 580 nm under photopic condition. Under mesopic condition, sensitivity in colour discrimination expanded to colour bands within the visible spectrum from 470 nm to 580 nm. Postoperative hue discrimination data under photopic condition are presented in Table 1; error scores of each patient are provided in Additional file 5. Data under mesopic condition are listed in Table 2; error scores of each patient are provided in Additional file 6.

### Changes of colour discrimination under mesopic condition

**Fig. 1:**
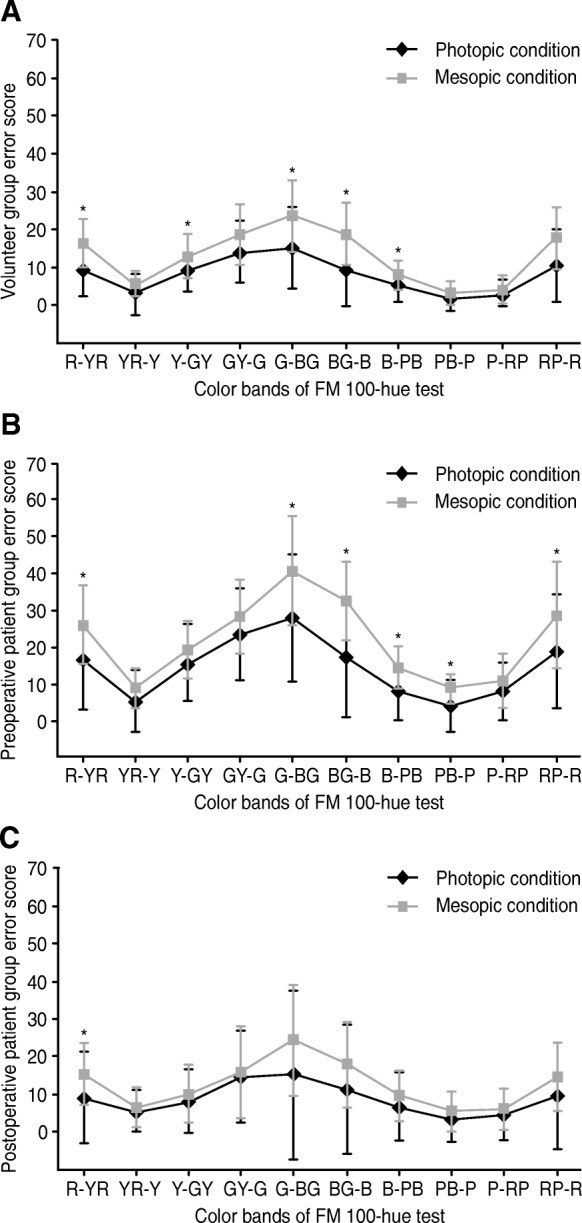
Hue discrimination under different illuminative levels. Colour perception was adversely affected by decreased illuminative level. Illuminative level of photopic and mesopic condition was 1000 lx and 40 lx, respectively. (**a**) In the control group, significant deterioration of hue perception under mesopic condition was found in bands of R-YR, Y-GY, G-BG, BG-B, B-PB and RP-R (*p* ≤ 0.002). (**b**) Preoperatively, the patient group manifested worse hue perception in colour bands of R-YR, G-BG, BG-B, B-PB, PB-P and RP-R (*p* ≤ 0.001). (**c**) In the postoperative phase, decline of hue perception was detected in the separate band of R-YR (*p* = 0.001). Error bars show standard deviation

Paired comparisons of error scores under mesopic condition and photopic condition were used to assess the effects of illuminative changes on colour discrimination. Under mesopic condition, increased TES were observed in the volunteer group and patient group for deteriorating general colour perception at both preoperative and postoperative phases (paired *t*-test, *p* ≤ 0.005). In the volunteer group (Fig. 1A), significant differences were found in the R-YR, Y-GY, G-BG, BG-B, B-PB and RP-R colour bands (paired *t*-test, *p* ≤ 0.002), corresponding with light wavelengths of 450 nm to 500 nm. Preoperatively, hue discrimination changes at different illumination levels indicated significant differences in PES in the patient group were detected in the R-YR, G-BG, BG-B, B-PB, PB-P and RP-R the colour bands (paired *t*-test, *p* ≤ 0.001, Fig. 1B), corresponding with light wavelengths ranging from the short-wavelength end to 500 nm. Postoperatively, only performances in the R-YR colour band manifested significant difference between the two illumination levels (paired *t*-test, *p* = 0.001, Fig. 1C). At low levels of illumination, these results indicated a severe colour perception decline in eyes with mild nuclear sclerosis to surface colours correlated with 500 nm to 450 nm. In eyes with age-related cataracts, this impact broadens to colours correlated with 500 nm to the short wavelength end. However, pseudophakic eyes maintained relatively stable hue perception under mesopic condition.

## Discussion

The aim of this study was to evaluate the impact of age-related cataracts on colour perception and recovery after phacoemulsification surgery. We also identified correlated spectra by comparing the surface colours of the Munsell system in the CIE diagram. Our results showed that colour discrimination was significantly affected by age-related cataracts. Cataract surgery effectively restored colour perception in the elderly; postoperative hue discrimination was comparable with volunteers approximately 8 years younger. The increase in photopic colour bands primarily corresponded with light wavelengths from 490 nm to 580 nm, which shifted to 470 nm to 580 nm under mesopic condition.

We employed the FM 100-hue test as a tool to evaluate the effects of age-related cataracts on hue discrimination and expanded the measurement scale to colour perception across the entire visible spectrum. For complex interactions between colour signals from the two eyes and the effects of colour constancy [3, 17], we recruited patients undergoing binocular cataract surgery and binocularly tested colour perception. To ensure the compatibility of visual signals between the two eyes, we required as inclusion criteria, compatible binocular visual acuity and lens opacity score. Compared with the volunteer group, postoperative colour vision enhancements were verified. When correlated with the CIE chromaticity diagram, the corresponding light wavelength spectra were determined.

Using minimum motion tasks [21] and potential latency measurements [8], previous studies have observed significant declines in sensitivity to monochromatic blue lights in elderly participants with mild nuclear sclerosis. Investigators have also analysed colour discrimination to certain colour axes by vector lengths when using the Cambridge Colour Test [6] and colour matching experiments [11]; profound age-related impacts on discrimination along the blue-yellow axis were reported. In our study, age-related cataracts profoundly impacted colour discrimination. Under photopic condition, the deterioration of colour perception primarily occurred for the continuous Y-GY, GY-G, G-BG and BG-B colour bands. Our data further confirmed the adverse effects of age-related cataracts on surface colour hue perception of yellow, greenish yellow, yellow green, greenish blue and blue colours along the blue-yellow axis. This result was consistent with previous studies and verified the similarity of physiological mechanisms between age-related cataracts [35] and nuclear sclerosis. In our study, the affected colour bands broadened to include yellowish green, green and bluish green surface colours associated with the red-green axis. This result indicate that cataracts significantly interfere with the contrast and saturation of retinal images, which are crucial factors affecting colour vision test performance for the elderly [11], and cause a more extensive hue sensitivity decline. According to correlation analysis on lens opacity and the error scores of FM 100-Hue, yellowing and lens nucleus opacity may be the main factors that impact colour perception. Colour perceptions of greenish blue and blue were more vulnerable to these changes.

Past studies have indicated that the ageing natural lens becomes a strong filter attenuating transmittance of all visible light, especially at short wavelengths (i.e., 400–550 nm) [36]. This filtering effect significantly increases after 60 years of age [37]. The transmittance decreases with age by 0.8–0.85% per year for blue light and 0.28–0.5% per year for green light [38, 39]. Reported decreasing values for light wavelengths from 400 nm to 550 nm ranged from 24 to 53% [36, 37, 40]. In the present study, age-related cataracts adversely affected hue discrimination corresponding to light wavelengths from 470 nm to 580 nm under photopic condition. Compared with normal ageing, the affected spectrum was skewed towards moderate wavelengths. The point at long-wavelengths of the skewness could be attributed to the severe attenuation of visible light by cataracts, which would then expand the affected spectrum range. Mild nuclear sclerosis in our control group could be an explanation for the point at the short-wavelengths of the skewness. Normal ageing lens in the control group leads to the decline of transmittance in the short wavelength range, which concealed differences in colour bands correlated with light wavelengths less than 470 nm.

The IOL effects on colour perception after cataract surgery has been measured using achromatic-point setting tests [19], colour contrast sensitivity tests along tritan axis [41] and minimum motion tasks for red-blue iso-illumination values [21]. The results of these studies, derived from patient groups implanted with UV light-filtering IOL, verified our observed significant increases in sensitivity along the yellow-blue axis after cataract surgery. However, no observable hue perception trend across the entire colour spectrum has been reported. In the present study, to avoid the effects of blue light-filtering IOL on perception along the blue-yellow axis or sensitivity to blue light spectrum under mesopic light condition [32, 42], we recruited patients implanted with UV light-filtering IOL in the patient group. PES analyses on the FM circuit revealed that postoperative photopic hue discrimination changes were most prominent in yellow, greenish yellow, yellow green, yellowish green, green and bluish green surface colours, which broadened to include greenish blue under mesopic condition. When compared with younger volunteers, our results confirmed colour perception benefits of cataract surgery. Past studies have also verified that with long-term adjustments and compensation to achieve colour constancy [10, 13, 17, 43], the ageing visual pathway remained sufficiently sensitive to discriminate subtle colour appearances as in younger visual systems. Unlike the prolonged course of age-related cataracts, phacoemulsification surgery instantly restored the transparency of ocular media but did not modulate the increased signals. This may be a key point in interpreting subjective experiences of colour appearance postoperative, which has been termed cyanopsia [22].

By observing chromatic renormalization, a large increase in the transmittance of lights with wavelengths below 500 nm after cataract surgery was verified [20]. Using the spot reflectometer technique, an improving factor of 4.0 has been reported for transmittance between 420 nm and 500 nm, whereas the average improvement was 1.3 between 500 nm and 750 nm [44]. In our study, the spectrum corresponding to postoperative colour vision improvement under photopic condition ranged from 490 nm to 580 nm, which shifted to 470 nm to 580 nm under mesopic condition. The improved colour vision after cataract surgery was a combined effect due to increased transmittance across the entire visible spectrum and filtered short-wavelength lights. Therefore, the affected spectra associated with the enhanced hue perception were not restricted to short-wavelengths; the direct measurements of light transmittance skewed towards the moderate-wavelengths.

Hue perception analyses under mesopic condition provided additional information on the transmitting property differences. At postoperative phase, in the patient group, no perception deterioration in continuous bands was observed. In the volunteer group with mild nuclear sclerosis, perception deterioration resulting from decreased illumination was restricted to colour bands associated with 450 nm to 500 nm wavelengths. At preoperative phase, in the patient group, for severe filtering and attenuating effects, impacts broadened to light wavelengths of 500 nm to short-wavelengths end. These results confirmed the differences between IOL and ageing lens in filtering light wavelengths below 500 nm [36–40]. To ensure a relatively stable hue perception under low illumination levels, an optimal IOL provides proper transmittance for light wavelengths longer than 450 nm.

There were several limitations in our study. The volunteer group was not exactly age-matched with the patient group; this may be a confounding factor in the study. However, the postoperative colour perception was comparable to that in the volunteer group, the average age of which was approximately 8 years younger. This demonstrated that lens ageing was the main factor in changes of colour vision as has been reported in a previous study [12]. In future studies, different age groups of young volunteers (40, 50 and 60-year-olds) and age-matched volunteer groups would be selected to explore postoperative colour vision changes and effects of age. Despite no significant differences in gender ratio, this factor remains a potential confounding factor. In future studies, a research design by gender grouping would be employed, and the differences in postoperative benefits resulting from gender difference could be investigated. Patients implanted with blue light-filtering IOL may be recruited in future studies, and differences in postoperative benefits in terms of colour vision would be compared with UV light-filtering IOL. The purpose of present study was to explore the impact of age-related cataracts on colour perception and subsequent recovery after cataract surgery; crystalline lens was only evaluated by grading LOCS-III scores. In future studies, quantification by Scheimpflug photography could be employed to illustrate the relationship between lens opacity and colour vision.

## Conclusions

Age-related cataracts severely impacted the colour vision of patients. Phacoemulsification surgery effectively restored colour perception. The impacts of lens opacity and postoperative benefits were most significant in colour bands correlated with light wavelengths from 470 nm to 580 nm. In future studies, a perceptual learning system targeting this spectrum could be developed to facilitate adaptions to sudden improvements in colour perception. In consideration of relatively stable hue perceptions at different illumination levels, a reasonable transmittance in spectra with wavelengths longer than 450 nm may be an important factor affecting postoperative colour discrimination.

## Additional files

Additional file 1: In a format of DOC, with a tile of Photopic TES and PES of the patient group at preoperative phase, describing the details of error scores of each patient at preoperative phase under photopic condition. (DOC 85 kb)

Additional file 2: In a format of DOC, with a tile of Mesopic TES and PES of the patient group at preoperative phase, describing the details of error scores of each patient at preoperative phase under mesopic condition. (DOC 87 kb)

Additional file 3: In a format of DOC, with a tile of Photopic TES and PES of the control group, describing the details of error scores of each volunteer in the control group under photopic condition. (DOC 87 kb)

Additional file 4: In a format of DOC, with a tile of Mesopic TES and PES of the control group, describing the details of error scores of each volunteer in the control group under mesopic condition. (DOC 87 kb)

Additional file 5: In a format of DOC, with a tile of Photopic TES and PES of the patient group at postoperative phase, describing the details of error scores of each patient at postoperative phase under photopic condition. (DOC 87 kb)

Additional file 6: In a format of DOC, with a tile of Mesopic TES and PES of the patient group at postoperative phase, describing the details of error scores of each patient at postoperative phase under mesopic condition. (DOC 88 kb)

## Abbreviations

BG-B: Blue-green to blue
B-PB: Blue to purple-blue
C: Cortical opalescence
CDVA: Corrected distant visual acuity
FM: Farnsworth-Munsell
G-BG: Green to blue-green
GY-G: Green-yellow to green
logMAR: Logarithm of the minimum angle of resolution
NC: Nuclear colour
NO: Nuclear opalescence
P: Posterior subcapsular opalescence
PB-P: Purple-blue to purple
PES: Partial error score
P-RP: Purple to red-purple
RP-R: Red-purple to red
R-YR: Red to yellow-red
SD: Standard deviations
TES: Total error score
Y-GY: Yellow to green-yellow
YR-Y: Yellow-red to yellow
