# Supplementary material for: The impact of age-related cataracts on colour perception, postoperative recovery and related spectra derived from test of hue perception
Source: BMC Ophthalmol. 2019 Feb 20;19:56. doi: 10.1186/s12886-019-1057-6 (PMC6383292; doi:10.1186/s12886-019-1057-6)
Supplement: Supplementary file 2 — In a format of DOC, with a tile of Mesopic TES and PES of the patient group at preoperative phase, describing the details of error scores of each patient at preoperative phase under mesopic condition. (DOC 87 kb) [file 12886_2019_1057_MOESM2_ESM.doc]

|  |  |  | **PES** | | | | | | | | | |
| --- | --- | --- | --- | --- | --- | --- | --- | --- | --- | --- | --- | --- |
| **Number of case** | **Group** | **TES** | **R-YR** | **YR-Y** | **Y-GY** | **GY-G** | **G-BG** | **BG-B** | **B-PB** | **PB-P** | **P-RP** | **RP-R** |
| 1 | patient-pre* | 128 | 15 | 5 | 9 | 31 | 36 | 23 | 15 | 5 | 0 | 11 |
| 2 | patient-pre* | 72 | 18 | 6 | 1 | 9 | 14 | 10 | 6 | 5 | 0 | 13 |
| 3 | patient-pre* | 80 | 11 | 4 | 3 | 9 | 24 | 20 | 7 | 0 | 0 | 12 |
| 4 | patient-pre* | 220 | 30 | 13 | 13 | 24 | 57 | 55 | 27 | 2 | 8 | 23 |
| 5 | patient-pre* | 220 | 33 | 20 | 24 | 29 | 48 | 31 | 7 | 16 | 12 | 23 |
| 6 | patient-pre* | 256 | 30 | 13 | 15 | 39 | 58 | 54 | 16 | 7 | 14 | 39 |
| 7 | patient-pre* | 204 | 33 | 15 | 25 | 38 | 24 | 21 | 10 | 16 | 14 | 33 |
| 8 | patient-pre* | 104 | 7 | 0 | 9 | 14 | 20 | 16 | 17 | 8 | 4 | 18 |
| 9 | patient-pre* | 112 | 17 | 6 | 21 | 24 | 28 | 12 | 1 | 4 | 0 | 9 |
| 10 | patient-pre* | 164 | 14 | 6 | 17 | 26 | 50 | 30 | 8 | 1 | 5 | 23 |
| 11 | patient-pre* | 280 | 31 | 31 | 37 | 41 | 46 | 53 | 25 | 13 | 11 | 44 |
| 12 | patient-pre* | 252 | 41 | 22 | 26 | 43 | 53 | 48 | 5 | 18 | 15 | 15 |
| 13 | patient-pre* | 300 | 46 | 20 | 35 | 45 | 62 | 51 | 22 | 11 | 14 | 50 |
| 14 | patient-pre* | 308 | 37 | 8 | 17 | 43 | 77 | 57 | 29 | 5 | 23 | 54 |
| 15 | patient-pre* | 232 | 34 | 5 | 4 | 18 | 56 | 57 | 23 | 6 | 20 | 46 |
| 16 | patient-pre | 177 | 28 | 8 | 6 | 14 | 52 | 51 | 13 | 4 | 7 | 20 |
| 17 | patient-pre | 100 | 0 | 0 | 15 | 21 | 18 | 21 | 8 | 2 | 10 | 16 |
| 18 | patient-pre | 272 | 50 | 25 | 26 | 32 | 47 | 21 | 20 | 6 | 26 | 46 |
| 19 | patient-pre | 196 | 23 | 0 | 10 | 20 | 45 | 35 | 15 | 10 | 17 | 40 |
| 20 | patient-pre | 144 | 18 | 0 | 16 | 18 | 25 | 26 | 6 | 12 | 11 | 33 |
| 21 | patient-pre | 228 | 23 | 8 | 36 | 43 | 34 | 25 | 9 | 14 | 29 | 41 |
| 22 | patient-pre | 196 | 20 | 0 | 25 | 42 | 53 | 31 | 5 | 0 | 10 | 31 |
| 23 | patient-pre | 316 | 47 | 1 | 20 | 37 | 66 | 55 | 24 | 20 | 21 | 56 |
| 24 | patient-pre | 48 | 4 | 0 | 8 | 8 | 11 | 5 | 8 | 0 | 4 | 4 |
| 25 | patient-pre | 256 | 44 | 10 | 38 | 43 | 60 | 36 | 8 | 9 | 6 | 25 |
| 26 | patient-pre | 224 | 33 | 1 | 28 | 33 | 44 | 53 | 17 | 8 | 2 | 40 |
| 27 | patient-pre | 272 | 36 | 14 | 29 | 49 | 33 | 23 | 26 | 22 | 19 | 49 |
| 28 | patient-pre | 124 | 0 | 2 | 29 | 23 | 20 | 14 | 20 | 8 | 6 | 13 |
| 29 | patient-pre | 176 | 26 | 13 | 22 | 18 | 27 | 23 | 22 | 28 | 13 | 7 |
| 30 | patient-pre | 176 | 33 | 14 | 16 | 17 | 36 | 24 | 15 | 9 | 7 | 27 |

**Additional file 2. Mesopic TES and PES of the patient group at preoperative phase.** TES, total error score of FM 100-hue test; PES, partial error score of the 10 color bands of FM 100-hue test; *, data cited in the calculation of sample size. R-YR= red to yellow-red; YR-Y= yellow-red to yellow; Y-GY= yellow to green-yellow; GY-G= green-yellow to green; G-BG= green to blue-green; BG-B= blue-green to blue; B-PB= blue to purple-blue; PB-P= purple-blue to purple; P-RP= purple to red-purple; RP-R= red-purple to red.
