# Supplementary material for: The impact of age-related cataracts on colour perception, postoperative recovery and related spectra derived from test of hue perception
Source: BMC Ophthalmol. 2019 Feb 20;19:56. doi: 10.1186/s12886-019-1057-6 (PMC6383292; doi:10.1186/s12886-019-1057-6)
Supplement: Supplementary file 3 — In a format of DOC, with a tile of Photopic TES and PES of the control group, describing the details of error scores of each volunteer in the control group under photopic condition. (DOC 87 kb) [file 12886_2019_1057_MOESM3_ESM.doc]

|  |  |  | **PES** | | | | | | | | | |
| --- | --- | --- | --- | --- | --- | --- | --- | --- | --- | --- | --- | --- |
| **Number of case** | **Group** | **TES** | **R-YR** | **YR-Y** | **Y-GY** | **GY-G** | **G-BG** | **BG-B** | **B-PB** | **PB-P** | **P-RP** | **RP-R** |
| 1 | control | 32 | 8 | 0 | 7 | 10 | 3 | 2 | 0 | 4 | 0 | 1 |
| 2 | control | 52 | 15 | 1 | 4 | 6 | 19 | 4 | 0 | 0 | 0 | 12 |
| 3 | control | 28 | 0 | 4 | 5 | 12 | 1 | 0 | 0 | 0 | 4 | 5 |
| 4 | control | 108 | 12 | 13 | 5 | 15 | 20 | 12 | 16 | 0 | 9 | 17 |
| 5 | control | 80 | 15 | 6 | 13 | 19 | 13 | 8 | 4 | 0 | 0 | 9 |
| 6 | control | 44 | 0 | 0 | 7 | 18 | 9 | 8 | 4 | 3 | 2 | 0 |
| 7 | control | 64 | 8 | 0 | 7 | 22 | 5 | 6 | 8 | 0 | 8 | 9 |
| 8 | control | 28 | 4 | 0 | 7 | 10 | 4 | 1 | 0 | 0 | 4 | 1 |
| 9 | control | 128 | 17 | 0 | 23 | 25 | 24 | 18 | 4 | 0 | 4 | 23 |
| 10 | control | 44 | 4 | 4 | 7 | 14 | 12 | 0 | 0 | 0 | 4 | 1 |
| 11 | control | 100 | 11 | 6 | 13 | 19 | 24 | 21 | 8 | 0 | 0 | 9 |
| 12 | control | 68 | 9 | 4 | 10 | 19 | 10 | 2 | 4 | 5 | 3 | 10 |
| 13 | control | 72 | 8 | 0 | 9 | 22 | 8 | 1 | 4 | 1 | 9 | 19 |
| 14 | control | 108 | 16 | 6 | 11 | 14 | 21 | 14 | 8 | 0 | 4 | 24 |
| 15 | control | 40 | 7 | 4 | 0 | 0 | 4 | 0 | 8 | 5 | 4 | 13 |
| 16 | control | 60 | 4 | 1 | 10 | 21 | 5 | 4 | 4 | 0 | 9 | 9 |
| 17 | control | 116 | 15 | 8 | 11 | 26 | 20 | 5 | 12 | 5 | 5 | 20 |
| 18 | control | 164 | 27 | 8 | 20 | 19 | 20 | 22 | 7 | 16 | 10 | 33 |
| 19 | control | 36 | 4 | 1 | 9 | 4 | 16 | 4 | 0 | 0 | 0 | 0 |
| 20 | control | 136 | 18 | 3 | 18 | 23 | 30 | 20 | 8 | 4 | 10 | 12 |
| 21 | control | 40 | 4 | 1 | 9 | 4 | 12 | 4 | 4 | 1 | 0 | 4 |
| 22 | control | 132 | 13 | 0 | 20 | 26 | 32 | 31 | 9 | 2 | 0 | 19 |
| 23 | control | 40 | 11 | 0 | 0 | 0 | 9 | 10 | 4 | 0 | 0 | 12 |
| 24 | control | 36 | 8 | 5 | 6 | 1 | 9 | 9 | 5 | 0 | 0 | 0 |
| 25 | control | 56 | 3 | 0 | 2 | 15 | 10 | 18 | 9 | 0 | 1 | 6 |
| 26 | control | 52 | 4 | 0 | 4 | 4 | 16 | 12 | 8 | 0 | 1 | 9 |
| 27 | control | 84 | 21 | 6 | 9 | 8 | 25 | 9 | 5 | 1 | 0 | 10 |
| 28 | control | 96 | 10 | 0 | 14 | 17 | 36 | 7 | 4 | 1 | 4 | 15 |
| 29 | control | 64 | 2 | 0 | 7 | 16 | 22 | 21 | 1 | 0 | 0 | 3 |
| 30 | control | 36 | 0 | 0 | 1 | 4 | 2 | 8 | 4 | 4 | 6 | 11 |

**Additional file 3. Photopic TES and PES of the control group.** TES, total error score of FM 100-hue test; PES, partial error score of the 10 color bands of FM 100-hue test. R-YR= red to yellow-red; YR-Y= yellow-red to yellow; Y-GY= yellow to green-yellow; GY-G= green-yellow to green; G-BG= green to blue-green; BG-B= blue-green to blue; B-PB= blue to purple-blue; PB-P= purple-blue to purple; P-RP= purple to red-purple; RP-R= red-purple to red.
